# Magnetic Resonance–Guided Focused Ultrasound Thalamotomy May Spare Dopaminergic Therapy in Early‐Stage Tremor‐Dominant Parkinson's Disease: A Pilot Study

**DOI:** 10.1002/mds.29200

**Published:** 2022-08-29

**Authors:** Nico Golfrè Andreasi, Roberto Cilia, Luigi Michele Romito, Salvatore Bonvegna, Giulia Straccia, Antonio Emanuele Elia, Alessio Novelli, Giuseppe Messina, Giovanni Tringali, Vincenzo Levi, Grazia Devigili, Sara Rinaldo, Valentina Gasparini, Marina Grisoli, Mario Stanziano, Francesco Ghielmetti, Sara Prioni, Elisa Bocchi, Paolo Amami, Sylvie Hélène Marie Jeanne Piacentini, Elisa Francesca Maria Ciceri, Maria Grazia Bruzzone, Roberto Eleopra

**Affiliations:** ^1^ Fondazione IRCCS Istituto Neurologico Carlo Besta, Department of Clinical Neurosciences Parkinson and Movement Disorders Unit Milan Italy; ^2^ Fondazione IRCCS Istituto Neurologico Carlo Besta, Neurosurgery Department Functional Neurosurgery Unit Milan Italy; ^3^ Fondazione IRCCS Istituto Neurologico Carlo Besta Neuroradiology Unit Milan Italy; ^4^ Neuroscience Department "Rita Levi Montalcini" University of Turin Turin Italy; ^5^ Fondazione IRCCS Istituto Neurologico Carlo Besta Health Department Milan Italy; ^6^ Fondazione IRCCS Istituto Neurologico Carlo Besta Clinical Neuropsychology Unit Milan Italy; ^7^ Fondazione IRCCS Istituto Neurologico Carlo Besta Diagnostic Radiology and Interventional Neuroradiology Milan Italy

**Keywords:** Parkinson's disease, tremor, thalamotomy, VIM, MRgFUS

## Abstract

**Background:**

Magnetic resonance–guided focused ultrasound (MRgFUS) thalamotomy is a safe and effective procedure for drug‐resistant tremor in Parkinson's disease (PD).

**Objective:**

The aim of this study was to demonstrate that MRgFUS ventralis intermedius thalamotomy in early‐stage tremor‐dominant PD may prevent an increase in dopaminergic medication 6 months after treatment compared with matched PD control subjects on standard medical therapy.

**Methods:**

We prospectively enrolled patients with early‐stage PD who underwent MRgFUS ventralis intermedius thalamotomy (PD‐FUS) and patients treated with oral dopaminergic therapy (PD‐ODT) with a 1:2 ratio. We collected demographic and clinical data at baseline and 6 and 12 months after thalamotomy.

**Results:**

We included 10 patients in the PD‐FUS group and 20 patients in the PD‐ODT group. We found a significant increase in total levodopa equivalent daily dose and levodopa plus monoamine oxidase B inhibitors dose in the PD‐ODT group 6 months after thalamotomy.

**Conclusions:**

In early‐stage tremor‐dominant PD, MRgFUS thalamotomy may be useful to reduce tremor and avoid the need to increase dopaminergic medications. © 2022 The Authors. *Movement Disorders* published by Wiley Periodicals LLC on behalf of International Parkinson and Movement Disorder Society

The thalamic nucleus ventralis intermedius (VIM) is a widely used target for the surgical treatment of medication‐resistant tremor in patients with essential tremor (ET) and Parkinson's disease (PD).^1^, ^2^ Recently, the ablative procedure gained renewed interest because of the introduction in clinical practice of magnetic resonance–guided focused ultrasound (MRgFUS), which allows to ablate deep brain structures through thermal coagulation without opening the skull.^3^

To date, MRgFUS VIM thalamotomy has been mostly focused on ET and proved to be safe and effective.^4^, ^5^ Studies on the effects of MRgFUS VIM thalamotomy in PD are, on the contrary, still scarce.^3^ To our knowledge, only one randomized clinical trial has been published, showing improvement of tremor lasting for 1 year.^6^ Overall, the outcome of MRgFUS thalamotomy has been described in less than 100 patients with tremor‐dominant PD.^7^, ^8^, ^9^, ^10^, ^11^, ^12^, ^13^, ^14^

Pharmacological treatment of tremor in PD may be challenging because this symptom may be levodopa resistant^15^ or show a “pseudoresistance” requiring higher dosages of dopaminergic medications than other cardinal symptoms.^16^ However, increasing dopaminergic medications may lead to adverse effects (AEs), such as increasing the risk for impulse control disorder (ICD)^17^ or motor fluctuations and dyskinesias.^18^, ^19^ To our knowledge, no study specifically investigated whether MRgFUS VIM thalamotomy may allow a sustained reduction of dopaminergic therapy dosage and thus be considered a modern “dopaminergic therapy–sparing strategy” in tremor‐dominant PD.

We hypothesized that MRgFUS thalamotomy may be performed at the earliest stages of tremor‐dominant PD aiming to “spare” dopaminergic medications and thus reduce the risk associated with the progressive increase of oral medical therapy over time.

In this study, the primary objective was to demonstrate that MRgFUS VIM thalamotomy performed in patients with early‐stage tremor‐dominant PD is associated with a significantly lower dopaminergic therapy daily dosage after 6 months compared with a matched PD control population on standard medical therapy.

## Subjects and Methods

We included patients diagnosed with early‐stage idiopathic PD^20^, ^21^ and tremor‐dominant clinical phenotype^22^ who underwent MRgFUS VIM thalamotomy (PD‐FUS) between February 1, 2019, and March 31, 2021, and with at least one follow‐up visit ≥6 months after the procedure. Early‐stage PD was defined as follows: total duration of dopaminergic therapy between 6 months and 4 years and no history of motor fluctuations or dyskinesias.^23^ MRgFUS eligibility criteria, which comprises the presence of medication‐refractory tremor, are detailed in Supporting Information Material 1.

Data from PD‐FUS were compared with those of a control group of patients with tremor‐dominant PD treated with oral dopaminergic therapy (PD‐ODT), matched by sex, age, and disease duration in a 1:2 ratio. In the PD‐ODT group, we included patients who met inclusion criteria but did not undergo MRgFUS.

All patients were visited by neurologists expert in movement disorders, who assessed motor performance (Movement Disorders Society Unified Parkinson's Disease Rating Scale‐motor Part III [MDS‐UPDRS‐III]) and Hoehn and Yahr stage in the morning 90 minutes after levodopa intake (*on* medication).^24^, ^25^

We collected demographical and clinical data at baseline, 6 months, and 12 months after surgery.

Dopaminergic drug therapy was calculated as levodopa equivalent daily dose (LEDD).^20^, ^26^ Total LEDD and LEDD associated separately with levodopa and monoamine oxidase B inhibitors (MAOB‐I) or dopamine agonists (DAs) were computed.

Change between baseline and follow‐up visits of MDS‐UPDRS‐III and LEDD were computed and expressed as percentage of change from baseline.

AEs were extensively collected for both groups; for PD‐FUS, we also collected AEs associated with thalamotomy and with MRI environment or ultrasonography procedure.^6^

MRgFUS VIM thalamotomy screening and procedure were approved by the local Ethics Committee (CE n.59/2020). All patients gave their written informed consent to the use of their anonymized clinical data for research purposes.

Details about the statistical analyses are reported in Supporting Information Material 1.

## Results

A total of 145 patients with tremor‐dominant PD were initially evaluated and referred to a dedicated outpatient clinic for the evaluation for MRgFUS thalamotomy. Eighty‐nine patients were accepted to undergo the full screening evaluations for MRgFUS VIM thalamotomy, of whom 37 underwent the procedure. Of these 37, we included 10 patients with early‐stage PD who fulfilled our a priori defined criteria.^21^ Among the 108 patients who were treated only with optimized drug therapy, 20 matched PD‐ODT were selected and included in the analysis. Details about the causes of exclusion are reported in Fig. S1.

Baseline demographic and clinical features were similar between PD‐FUS and PD‐ODT (Table 1).

**TABLE 1:** Baseline demographics and clinical characteristics and 6‐month follow‐up (PD‐FUS, n = 10; PD‐ODT, n = 20)

	Baseline		P value^a^ (differences at baseline)	6 months		Change from baseline (%)^b^		P value^c^
	PD‐FUS	PD‐ODT		PD‐FUS	PD‐ODT	PD‐FUS	PD‐ODT	
Demographic characteristics								
Sex (M/F)	8/2	16/4						
Age (y)	62.3 (60.2; 72.3)	62.87 (59.5; 72.1)	0.895					
Disease duration (y)	3.8 (2.4; 4.5)	3.2 (2.8; 4 0.1)	0.936					
Time to surgery (mo)	2.5 (1.7; 4.2)	NA						
Time to follow‐up visit (mo)				6.3 (5.0; 6.7)	7.4 (5.5; 8.6)			
Motor outcome (*on* medication)								
MDS‐UPDRS‐III total score^d^	22.5 (17.0; 28.0)	27.8 (20.3; 32.9)	0.102	15.5 (10.0; 20.0)	23.9 (18.5; 29.9)	−34.4 (−50.0; −12.0)	−18.0 (−22.9; 2.0)	**0.003**
Tremor^d^	8.0 (7.0; 9.8)	8.0 (6.0; 11.3)	0.691	3.0 (1.5; 4.8)	7.0 (5.0; 10.3)	63.3 (55.2; 82.5)	3.8 (−2.7; 25.0)	**<0.001**
Rigidity^d^	2.0 (2.0; 3.0)	4.0 (3.0; 6.3)	**0.012**	0.5 (0.0; 2.0)	2.5 (1.0; 5.0)	90.0 (17.9; 100.0)	25.0 (0.0; 66.7)	0.196
Bradykinesia^d^	6.5 (4.5; 8.75)	8.0 (7.0; 12.0)	0.069	6.0 (3.0; 6.8)	8.0 (5.8; 11.0)	0.0 (0.0; 22.3)	15.9 (0.0; 28.6)	0.534
H&Y^26^	2 (1; 2)	2 (1; 2)	1	2 (1; 2)	2 (2; 2)	0 (0; 0)	0 (0; 0)	0.235
Dopaminergic medications								
Duration (y)	1.9 (1.3; 2.6)	2.5 (2.2; 3.4)	0.148					
Total LEDD (mg/d)^e^	472.5 (300.0; 650.0)	400.0 (285.0; 525.0)	0.897	497.5 (300.0; 600.0)	527.5 (406.3; 632.5)	0.0 (−20.6; 16.0)	24.7 (9.0; 65.2)	**0.017**
Levodopa + MAOB‐Is dose (mg/d)^e,f^	350.0 (100.0; 500.0)	275.0 (100.0; 500.0)	0.756	325.0 (200.0; 462.5)	375.0 (250.0; 575.0)	0.0 (−12.5; 33.3)	17.5 (0.0; 50.0)	0.400
Patients on levodopa and/or MAOB‐Is, n (%)	7 (70%)	18 (90%)	0.300	8 (80%)	19 (95%)			
DA dose (mg/d)^e^	165.0 (150.0; 360.0)	120.0 (75.0; 300.0)	0.069	150.0 (120.0; 300.0)	120.0 (80.0; 240.0)	−10.00 (−33.3; 0.0)	0.0 (0.0; 0.0)	0.051
Patients on DA, n (%)	10 (100%)	14 (70%)	0.074	10 (100%)	17 (85%)			

Data are expressed as median (interquartile range) unless otherwise specified.

^a^ Differences at baseline were analyzed with unpaired *t* test or Mann–Whitney *U* test according to normality of the data.

^b^ Positive percent values represent an increase in score or dosage from baseline.

^c^ Analysis of the differences in the change from baseline variable between PD‐FUS and PD‐ODT; data were analyzed with analysis of variance corrected for baseline value or Mann–Whitney *U* test according to normality of the data. Significant data (*p* < 0.05) are shown in bold.

^d^ Motor outcome expressed as total MDS‐UPDRS‐III tremor score (sum of items 3.15, 3.16, 3.17, and 3.18), rigidity score (item 3.3), and bradykinesia score (sum of items 3.4–3.8).^25^

^e^LEDD was calculated according to Schade et al^20^ and Charles et al.^21^

^f^ Including both levodopa and monoamine oxidase B inhibitors.

PD‐FUS, patients with Parkinson's disease treated with magnetic resonance–guided focused ultrasound; PD‐ODT, patients with Parkinson's disease treated with oral dopaminergic therapy; M, male; F, female; MDS‐UPDRS‐III, Movement Disorder's Society Unified Parkinson's Disease Rating Scale–motor Part III; H&Y, Hoehn and Yahr stage; LEDD, levodopa equivalent daily dose; MAO‐I, monoamine oxidase inhibitor; DA, dopamine agonist.

**FIG. 1:**
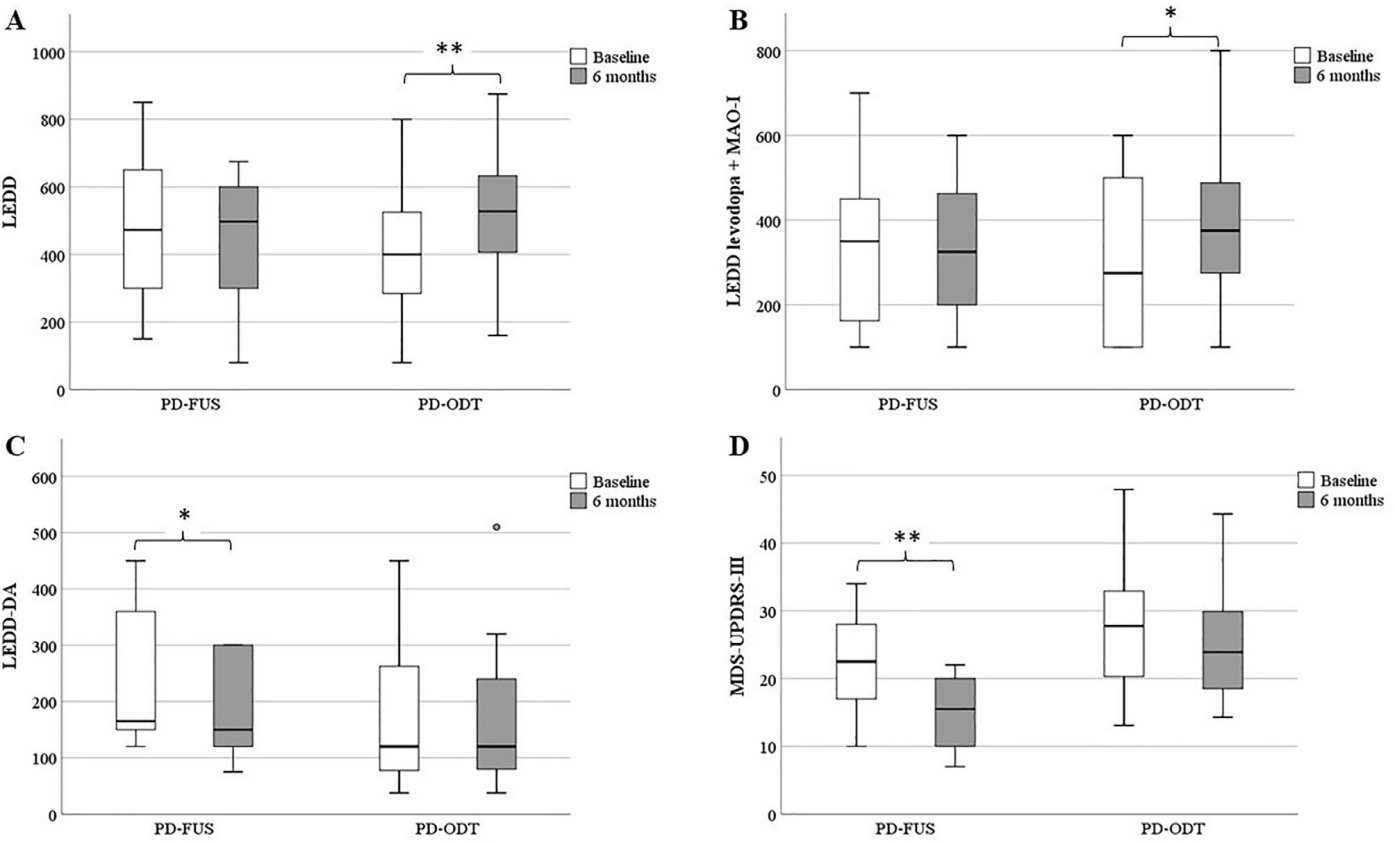
Change in dopaminergic medications and motor outcome between baseline and 6‐month follow‐up in patients with Parkinson's disease (PD) treated with magnetic resonance–guided focused ultrasound (PD‐FUS; n = 10) and patients with PD treated with oral dopaminergic therapy (PD‐ODT, n = 20). Dopaminergic medications are reported as levodopa equivalent daily dose (LEDD) and divided as total LEDD (**A**), LEDD of levodopa plus monoamine oxidase inhibitors (MAO‐Is) (**B**) and LEDD of dopamine agonists (LEDD DAs) (**C**). The motor outcome (**D**) is expressed with the Movement Disorders Society Unified Parkinson's Disease Rating Scale‐motor Part III (MDS‐UPDRS‐III; ranging from 0 to 132, with greater scores indicating greater severity). Paired Student *t* test or Wilcoxon test was applied depending on normality of the data. *P < 0.05; **P < 0.01.

At 6‐month follow‐up, we observed a significant difference in total LEDD between the two groups, because of increased LEDD in the PD‐ODT group versus stable dosage in PD‐FUS (Table 1, Fig. 1A).

We found a significant increase in total LEDD and LEDD of levodopa plus MAOB‐I in the PD‐ODT group (*P* = 0.001 and *P* = 0.014) and a mild reduction in LEDD of DA in the PD‐FUS group (*P* = 0.042) (Fig. 1A–C).

In both groups, we observed a reduction in MDS‐UPDRS‐III motor score at the 6‐month follow‐up (Fig. 1, Table 1). However, the change was significantly higher in PD‐FUS than PD‐ODT, which was due to improvement in tremor in the former group (Table 1). Notably, the reduction in MDS‐UPDRS‐III between baseline and 6‐month follow‐up was statistically significant only for the PD‐FUS group (*P* = 0.002), while in the PD‐ODT group there was only a trend to a statistically significant difference (*P* = 0.081) (Fig. 1D).

At 12‐month follow‐up, we found similar results despite the much‐limited sample size (PD‐FUS, n = 4; PD‐ODT, n = 8). Details are reported in Table S1 and Fig. S2. Indeed, total LEDD change was still significantly different between the two groups (*P* = 0.01), with a significant increase in total LEDD and LEDD of levodopa *plus* MAOB‐I in the PD‐ODT group after 12 months compared with baseline (*P* = 0.005 and *P* = 0.042, respectively) (Fig. S2A,B).

### Safety

AEs are detailed in Table S2. No serious AEs (ie, associated with new or prolonged hospitalization, permanent disability, or death) were found in either PD‐FUS or PD‐ODT; overall, no statistically significant differences were found in the AEs profile between PD‐FUS and PD‐ODT.

## Discussion

MRgFUS thalamotomy is a safe and effective treatment for tremor in patients with PD.^6^ In the present pilot study, our findings suggest that MRgFUS thalamotomy performed in patients with early‐stage tremor‐dominant PD allows to maintain low daily dosage of oral dopaminergic medications along with a good control of tremor for at least 6 months.

Management of dopaminergic therapy has never been specifically investigated by studies reporting data on MRgFUS thalamotomy in PD. In a recent clinical trial, Bond et al^6^ found a tendency to decrease dopaminergic medication in patients with PD treated with MRgFUS thalamotomy compared with an increase in patients who underwent the sham procedure; however, this trial was not designed to investigate this phenomenon, and the authors do not comment on this finding. Recently, Sinai et al^13^ suggested that MRgFUS VIM thalamotomy may delay initiation of levodopa treatment, and Yamamoto et al^14^ found a stabilization or increase in dopaminergic therapy after 1‐year follow‐up. In other studies, change in LEDD after MRgFUS VIM thalamotomy was not reported.^9^, ^10^, ^12^, ^27^ A previous study on stereotactic thalamotomy found a dramatic and long‐lasting reduction in dopaminergic medication^28^; more than half of these patients were, however, on Hoehn and Yahr stage III or IV, and direct comparison with our early‐stage PD population may be unreliable.

We found a stabilization of LEDD in PD‐FUS after VIM thalamotomy; differences in the study population, such as higher mean disease duration, motor score, and LEDD in the study of Bond et al,^6^ may explain the different results. Moreover, in early‐stage PD, it has been already shown that even deep brain stimulation of the subthalamic nucleus may not allow reduction of dopaminergic medications,^23^, ^29^ as opposed to deep brain stimulation of the subthalamic nucleus in patients with PD with motor complications.^30^, ^31^ It is therefore conceivable that unilateral VIM thalamotomy, which mainly improves tremor and no other cardinal features, may not allow dopaminergic therapy reduction in an early stage of the disease. The slight reduction in LEDD‐DA dose in the PD‐FUS group may not be clinically significant; for example, it has been shown that management for ICD required, on average, a reduction of more than 50% of the dosage of DAs.^32^ In an available trial on interventional therapy in patients with early‐stage PD, LEDD was increased during the study period in patients treated with only dopaminergic medications,^29^ as we observed in our study. Effective control of tremor in PD often requires higher doses of dopaminergic medication than rigidity and bradykinesia. Our data confirm that patients with early tremor‐dominant PD are likely to be treated with an increasingly high levodopa dose in an attempt to control their disabling tremor, with poor benefit. Dopaminergic medication can have behavioral side effects,^17^ and a higher cumulative levodopa exposure has been linked to the development of motor fluctuations and dyskinesias.^19^ We can speculate that treatments that improve motor symptoms without the need to increase dopaminergic therapy may delay or reduce the incidence of these side effects.^17^, ^33^

In all studies, the MDS‐UPDRS‐III score decreased significantly shortly after the procedure (eg, 1 month^6^, ^9^) with an improvement lasting for 3,^6^, ^10^ 6,^9^ and 12 or more months.^9^, ^13^, ^27^ In our study, the reduction in the total motor score, despite being clinically and statistically significant, appears to be inferior to that previously reported; in previous works, a nearly 50% reduction in the total UPDRS motor score after the procedure was noted,^6^, ^9^, ^10^, ^27^ while we noted a median 34.4% improvement in the MDS‐UPDRS‐III score. The use of a different scale, in which more points are attributed to different aspects of tremor, and the differences in the study populations may explain this finding.

AEs of MRgFUS VIM thalamotomy in our population are similar to what was previously reported^6^ and confirm the overall safety of this procedure.

Our findings suggest that MRgFUS VIM thalamotomy may result in better tremor control than optimized medical therapy, with an acceptable safety profile in patients at early disease stages presenting with unsatisfactory response to therapeutic dose of pharmacological strategies.

We acknowledge that our pilot study is limited by the small sample size and a short follow‐up; moreover, given the very limited number of patients who reached 12‐month follow‐up, the results on the long‐term management of dopaminergic medication after MRgFUS thalamotomy should be interpreted with caution. Finally, it would have been interesting to evaluate both groups in a defined *off* medication condition. Nevertheless, this cohort was at early‐stage PD, and none of those on levodopa had motor fluctuations at baseline probably because of the long‐duration response to levodopa.^34^ Therefore, a true *off* state would have required longer washout of all dopaminergic drugs than standard 12‐hour overnight withdrawal (lasting for several days), which was not performed for ethical reasons. However, there are strengths worth mentioning. Our strict selection and matching criteria, as well as the clinical homogeneity of the study population, provided statistically significant results despite the limited sample size. Further studies in larger cohorts with longer follow‐up are needed to confirm whether early MRgFUS may be a cost‐effective therapeutic strategy in early‐stage tremor‐dominant PD, potentially reducing the risk for AEs caused by the progressive increase of dopaminergic medications, such as motor complications and/or ICDs.

## Conclusion

In patients with early‐stage tremor‐dominant PD, MRgFUS thalamotomy may be useful to reduce tremor and avoid, in the short term, the need to increase dopaminergic medications. These results may help to understand the correct timing to address patients for MRgFUS thalamotomy, a treatment that, at the time being, should be reserved to patients with proven medication‐refractory tremor.

Prospective studies with larger cohorts are needed to confirm these findings and to understand whether this treatment may reduce the incidence of AEs and long‐term motor complications of dopaminergic therapy. Longer follow‐up may additionally provide helpful information on the difference in time to dyskinesias between the two groups.

## Author Roles

1. Research project: A. Conception, B. Organization, C. Execution;

2. Statistical Analysis: A. Design, B. Execution, C. Review and Critique;

3. Manuscript Preparation: A. Writing of the first draft, B. Review and Critique.

N.G.A.: 1B, 1C, 2A, 2B, 3A, 3B.

R.C.: 1A, 1B, 1C, 2A, 2C, 3A, 3B.

L.M.R.: 1C, 2C, 3B.

S.B.: 1B, 1C, 2C, 3B.

G.S.: 1B, 1C, 2C, 3B.

A.E.E.: 1B, 2C, 3B.

A.N.: 1B, 1C, 2C, 3B.

G.M.: 1B, 1C, 2C, 3B.

G.T.: 1B, 1C, 2C, 3B.

V.L.: 1B, 1C, 2C, 3B.

G.D.: 1B, 1C, 2C, 3B.

S.R.: 1B, 1C, 2C, 3B.

V.G.: 1C, 2B, 3A, 3B.

M.G.: 1B, 1C, 2C, 3B.

M.S.: 1B, 1C, 2C, 3B.

F.G.: 1B, 2C, 3C.

S.P.: 1C, 2C, 3B.

E.B.: 1B, 1C, 2B, 3B.

P.A.: 1B, 1C, 2B, 3B.

S.H.M.J.P.: 1B, 1C, 2C, 3B.

E.F.M.C.: 1B, 1C, 2C, 3B.

M.G.B.: 1B, 1C, 2C, 3B.

R.E.: 1A, 1B, 1C, 2A, 2C, 3B.

## Financial Disclosures of All Authors (for the Preceding 12 Months)

N.G.A., R.C., L.M.R., S.B., G.S., A.E.E., A.N., G.M., G.T., V.L., G.D., S.R., V.G., M.G., M.S., F.G., S.P., E.B., P.A., S.H.M.J.P., E.F.M.C., M.G.B., and R.E. have no financial disclosures to report.

## Financial Disclosures

R.C. has received speaking honoraria from Zambon; Zambon SAU; Bial Italia Srl; Advisory board fees from Bial; Research support from the Italian Ministry of Health; Editor‐in‐chief of the neuromuscular and movement disorders section of Brain Sciences; Member of the editorial board of Parkinsonism and related disorders, Frontiers in Neuroscience ‐ Neurodegeneration (Associate Editor) and Frontiers in Neurology ‐ Movement Disorders (Review Editor).

## Supporting information


**Appendix S1.** Supporting information

**Figure S1.** flow‐chart with description of the causes of exclusion and inclusion of patients.

**Figure S2.** change in dopaminergic medications and motor outcome at baseline, 6 and 12‐months in PD‐FUS (n = 4) and PD‐ODT‐ (n = 8) patients with 12‐months follow‐up.

**Table S1.** baseline demographics and clinical characteristics and 12‐months of patients with 12‐months follow up (PD‐FUS, n = 4; PD‐ODT, n = 8).

**Table S2.** characterization of incident Adverse Events observed during the 6‐months follow‐up.

## Data Availability

The data that support the findings of this study are available from the corresponding author upon reasonable request.
