# Supplementary material for: Magnetic Resonance–Guided Focused Ultrasound Thalamotomy May Spare Dopaminergic Therapy in Early‐Stage Tremor‐Dominant Parkinson's Disease: A Pilot Study
Source: Mov Disord. 2022 Aug 29;37(11):2289–95. doi: 10.1002/mds.29200 (PMC9804690; doi:10.1002/mds.29200)
Supplement: Supplementary file 1 — Appendix S1. Supporting information [file MDS-37-2289-s003.docx]

**Supplementary Material 1 - Criteria for eligibility to MRgFUS thalamotomy**

All the patients had a clinically significant tremor with unsatisfactory effect of dopaminergic or anticholinergic medications; all patients experienced embarrassment and social stigma due to tremor.

All the patients with Parkinson’s Disease were evaluated in the defined OFF-medication (at least 12 hours-withdrawal of any anti-parkinsonian medication) and ON-medication (90 minutes after a levodopa loading dose approximately equal to 150% of the patients’ usual morning dose of dopaminergic medication) conditions with the Movement Disorder Society Unified Parkinson’s Disease rating scale motor part (MDS-UPDRS-III). Patients with unsatisfactory improvement between ON- and OFF-medication conditions in the sum of tremor subscales were considered to have a medication resistant tremor.

All the patients had no contraindications for the procedure including:

- cognitive decline evaluated with extensive neuropsychological testing as advised by the Core Assessment Program for Surgical Interventional Therapies in Parkinson’s Disease (CAPSIT-PD) [1];
- unstable or severe psychiatric conditions like anxiety or depression disorder [1];
- alcohol or substances abuse as defined by the Diagnostic and Statistical Manual of Mental Disorders, fifth edition (DSM-5);
- history of intracranial haemorrhage, ischaemic stroke or neoplasms;
- intracranial aneurysms or arteriovenous malformations requiring treatment;
- anticoagulant or anti-platelet therapy which the patients were not allowed to interrupt;
- significant unstable medical conditions;
- overall skull density ratio <0.35 as calculated from the screening computed tomography.
- contraindications for MRI, including claustrophobia.

1. Defer G-L, Widner H, Marié R-M, et al (1999) Core assessment program for surgical interventional therapies in Parkinson’s disease (CAPSIT‐PD). Mov Disord 14:572–584. [https://doi.org/10.1002/1531-8257(199907)14:4<572::aid-mds1005>3.0.co;2-c](https://doi.org/10.1002/1531-8257(199907)14:4%3c572::aid-mds1005%3e3.0.co;2-c)

**Supplementary Material 1 - Statistical analysis**

Data normality was evaluated with the Shapiro-Wilk test. Baseline demographic and clinical characteristics between PD-FUS and PD-ODT were compared with t-student test or the Mann-Whitney-U test as appropriate. Comparison between longitudinal data was done with paired t-student test or Wilcoxon test as appropriate.

Changes in MDS-UPDRS-III and LEDD between baseline and follow-up visits were analysed by means of Analysis of Variance (ANOVA) or with the Mann-Whitney-U test. For ANOVA, analysis was adjusted for the baseline value of the considered variable.

All p values <0.05 were considered statistically significant.

Statistics were computed with SPSS Statistics v.25.0 (IBM, Armonk, New York, USA).
