# Supplementary material for: Magnetic Resonance–Guided Focused Ultrasound Thalamotomy May Spare Dopaminergic Therapy in Early‐Stage Tremor‐Dominant Parkinson's Disease: A Pilot Study
Source: Mov Disord. 2022 Aug 29;37(11):2289–95. doi: 10.1002/mds.29200 (PMC9804690; doi:10.1002/mds.29200)
Supplement: Supplementary file 2 — Figure S1. flow‐chart with description of the causes of exclusion and inclusion of patients. [file MDS-37-2289-s002.docx]

**Supplementary Figure 1:** flow-chart with description of the causes of exclusion and inclusion of patients.

89 accepted to undergo the screening evaluations for MRgFUS thalamotomy

~~52~~ 37 patients excluded for treatment:

- 9 patients with cognitive deficits;

- 5 patient with claustrophobia;

- 3 patients shifted to DBS;

- 3 patients with mild not disabling tremor;

- 2 patients SDR<0.35;

- 2 patients had a satisfactory benefit after optimization of oral therapy;

- 1 patient with atypical parkinsonism;

- 1 patient with pacemaker;

- 9 patients lost to follow up;

15 patients had no contraindications, but refused to undergo MRgFUS thalamotomy for various reasons:

- 11 were afraid of complications;

- 2 because the treatment required hospitalization and subsequent mandatory radiological and clinical follow-up;

- 2 patients refused for personal unknown reasons.

37 patients underwent MRgFUS thalamtomy

**10 patients (8 men, 2 women) included in the PD-FUS group**

- 22 patients with dopaminergic therapy duration <6 months or >48 months

- 2 patients with motor fluctuation at baseline

- 1 patient lost to follow up after 1 month follow up from MRgFUS thalamotomy

- 2 patients did not reach 6 months follow up

145 tremor-dominant PD patients evaluated in our outpatient clinics were asked for the willingness to treat tremor with MRgFUS thalamotomy

**20 matched patients (16 men, 4 women) included in the PD-ODT group**

1 patient with SDR<0.35

1 patient with claustrophobia;

4 patients who refused thalamotomy
