# Supplementary material for: Magnetic Resonance–Guided Focused Ultrasound Thalamotomy May Spare Dopaminergic Therapy in Early‐Stage Tremor‐Dominant Parkinson's Disease: A Pilot Study
Source: Mov Disord. 2022 Aug 29;37(11):2289–95. doi: 10.1002/mds.29200 (PMC9804690; doi:10.1002/mds.29200)
Supplement: Supplementary file 3 — Figure S2. change in dopaminergic medications and motor outcome at baseline, 6 and 12‐months in PD‐FUS (n = 4) and PD‐ODT‐ (n = 8) patients with 12‐months follow‐up. [file MDS-37-2289-s001.docx]

| **Supplementary Figure 2:** change in dopaminergic medications and motor outcome at baseline, 6 and 12-months in PD-FUS (n=4) and PD-ODT- (n=8) patients with 12-months follow-up.  Dopaminergic medications are reported as Levodopa Equivalent Daily Dose and divided as total LEDD (**A**), LEDD of levodopa plus Mono-Amino-Oxidase Inhibitors (MAO-I) (**B**) and LEDD of Dopamine Agonists (LEDD-DA) (**C**). The motor outcome (**D**) is expressed with the Movement Disorders Society Unified Parkinson’s Disease Rating Scale Motor Part (MDS-UPDRS-III; ranging from 0 to 132, with greater scores indicating greater severity).  Analysis between different time points were computed with Friedmann’s test; Bonferroni correction was applied for multiple comparisons.  *, p<0.05; **, p<0.01. |
| --- |
| 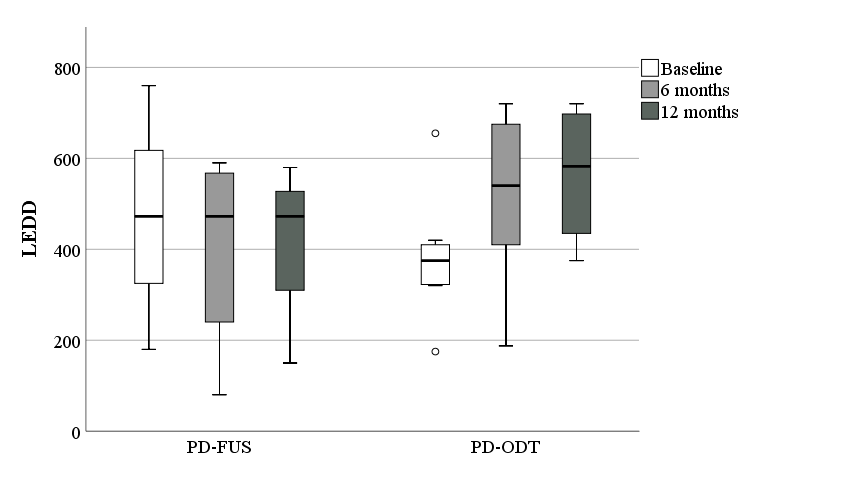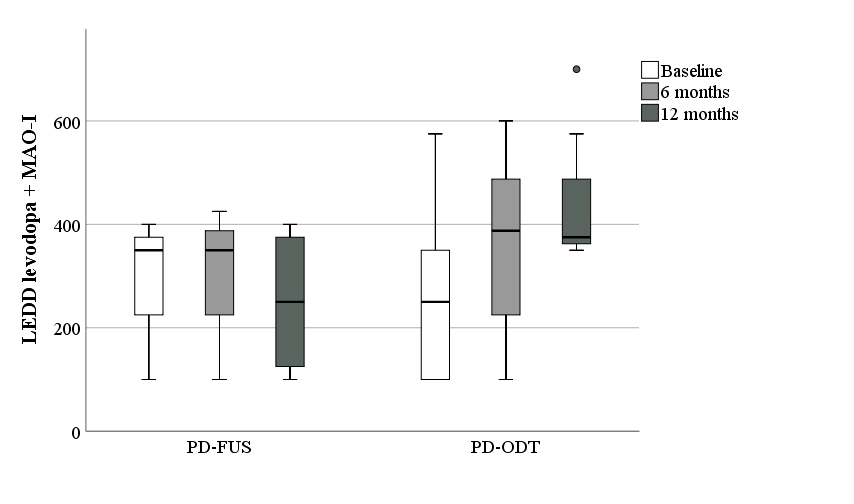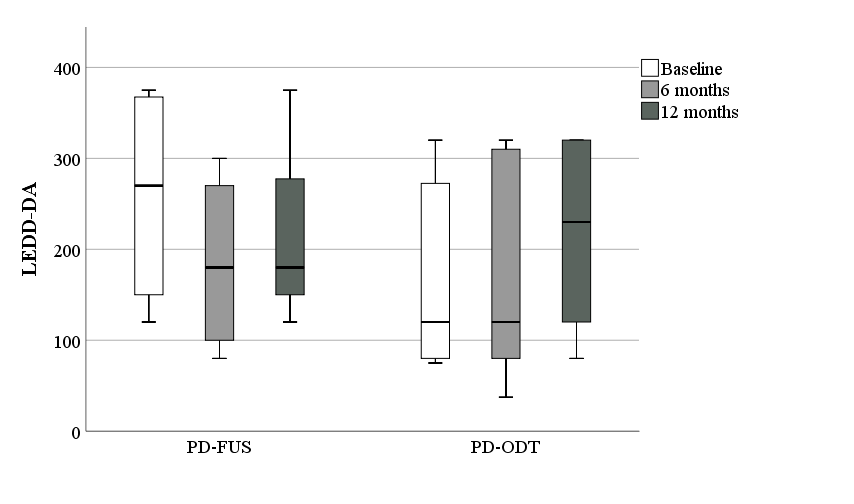 **A**  **B**  **C**  * 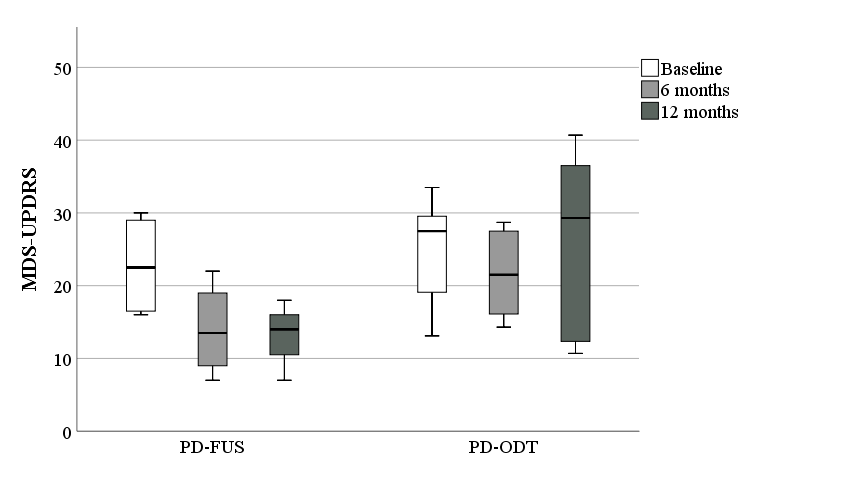 **-III**  **D**  ** |
