# Supplementary material for: Magnetic Resonance–Guided Focused Ultrasound Thalamotomy May Spare Dopaminergic Therapy in Early‐Stage Tremor‐Dominant Parkinson's Disease: A Pilot Study
Source: Mov Disord. 2022 Aug 29;37(11):2289–95. doi: 10.1002/mds.29200 (PMC9804690; doi:10.1002/mds.29200)
Supplement: Supplementary file 4 — Table S1. baseline demographics and clinical characteristics and 12‐months of patients with 12‐months follow up (PD‐FUS, n = 4; PD‐ODT, n = 8). [file MDS-37-2289-s005.docx]

| **Supplementary table 1:** baseline demographics and clinical characteristics and 12-months of patients with 12-months follow up (PD-FUS, n = 4; PD-ODT, n = 8). | | | | | | | | |
| --- | --- | --- | --- | --- | --- | --- | --- | --- |
|  | **Baseline** | | **p value** ^e^  **(differences at baseline)** | **12 months** | | **Change from baseline %** ^f^ | | **p value** ^e^  **(Change from baseline)** |
|  | **PD-FUS** | **PD-ODT** |  | **PD-FUS** | **PD-ODT** | **PD-FUS** | **PD-ODT** |  |
| **Demographic characteristics** | | | | | | | | |
| **Sex (M/F)** | 3/1 | 6/2 |  |  |  |  |  |  |
| **Age (years)** | 66.3  (60.6; 72.5) | 67.6  (60.1; 72.1) | 1 |  |  |  |  |  |
| **Disease Duration (years)** | 4.1 (2.9; 4.8) | 3.4 (2.3; 4.7) | .734 |  |  |  |  |  |
| **Time to surgery (months)** | 3.2 (1.4; 5.2) | NA |  |  |  |  |  |  |
| **Time to follow-up visit (months)** |  |  |  | 12.7  (12.1; 13.5) | 15.5  (13.5; 17.6) |  |  |  |
| **Motor outcome** (ON medication) | | | | | | | | |
| **MDS-UPDRS-III total score** ^a^ | 22.5  (16.5; 29.0) | 27.5  (19.1; 29.6) | .670 | 14.0  (10.5; 16.0) | 29.3  (12.4; 36.5) | -45.0  (-54.4; -26.3) | 14.3  (-30.9; 37.5) | .126 |
| **Tremor** ^a^ | 8.0 (6.0; 9.5) | 8.0 (6.0; 11.3) | .733 | 1.5 (1.0; 2.5) | 8.5 (6.0; 10.8) | 74.2 (63.9; 82.8) | 0.0 (-12.5; 3.3) | **.008** |
| **Rigidity** ^a^ | 3.5 (2.0; 5.5) | 3.5 (3.0; 4.3) | .999 | 1.5 (0.8; 2.3) | 3.5 (1.8; 6.3) | 68.6 (42.9; 85.0) | 0.0 (-100.0; 48.75) | .17 |
| **Bradykinesia** ^a^ | 7.0 (4.8; 8.3) | 7.0 (6.5; 8.5) | .732 | 4.5 (2.8; 6.3) | 9.5 (3.8; 13.5) | 4.2 (-37.5; 35.4) | -2.5 (-75.0; 35.0) | .933 |
| **HY** ^b^ | 2 (2; 2) | 2 (1; 2) | .097 | 2 (2; 2) | 2 (1; 3) | 0.0  (-25.0; 0.0) | 0.0  (0.0; 50.0) | .085 |
| **Dopaminergic medications** | | | | | | | | |
| **Duration (years)** | 1.6 (1.3; 2.8) | 2.3 (2.2; 2.8) | .395 |  |  |  |  |  |
| **Total LEDD (mg/day)^c^** | 472.5  (325.0; 617.5) | 375.0  (322.5; 410.0) | .234 | 472.5  (310.0; 527.5) | 582.5  (435.0; 697.5) | -8.3  (-20.2; 0.0) | 49.8  (25.1; 94.6) | **.010** |
| **Levodopa+MAO-I dose (mg/day) ^c,d^** | 350.0  (100.0; 400.0) | 250.0  (100.0; 400.0) | .723 | 250.0  (125.0; 375.0) | 375.0  (350.0; 575.0) | 0.0  (-12.5; 21.4) | 33.3  (0.0; 50.0) | .132 |
| **Patients on Levodopa and/or MAO-I n (%)** | 3 (75.0%) | 7 (87.5%) |  | 4 (100%) | 7 (87.5%) |  |  |  |
| **DA dose (mg/day)^c^** | 270.0  (150.0; 367.5) | 120.0  (80.0; 320.0) | .154 | 180.0  (120.0; 375.0) | 230.0  (120.0; 320.0) | 0.0  (-25.0; 0.0) | 0.0  (0.0; 50.0) | .085 |
| **Patients on DA n (%)** | 4 (100%) | 6 (75.0%) |  | 3 (75%) | 6 (75.0%) |  |  |  |
| Data expressed as median (interquartile range) unless otherwise specified.  Abbreviations: PD-FUS, Parkinson’s Disease patients treated with Magnetic Resonance guided Focused Ultrasound; PD-ODT, Parkinson’s Disease patients treated with Oral Dopaminergic Therapy; MDS-UPDRS-III, Movement Disorder’s Society Unified Parkinson’s Disease Rating Scale Motor score. HY: Hoehn Yahr Stage; LEDD, Levodopa Equivalent Daily Dose; MAO-I , Mono amino Oxidase Inhibitors; DA, Dopamine-Agonists.  ^a^, motor outcome expressed as total MDS-UPDRS-III, Tremor score (sum of items 3.15, 3.16, 3.17 and 3.18), Rigidity score (item 3.3) and Bradykinesia score (sum of items 3.4, 3.5, 3.6, 3.7, 3.8); Goetz CG et al. Mov Disord 2008;23:2129–2170.  ^b^, Hoehn MM, Yahr MD. Neurology 1967; 17:427–427.  ^c^, LEDD was calculated accordingly to Tomlinson CL et al . Mov Disord 2010; 25:2649–2653.  ^d^, including both Levodopa and Monoamino Oxidase type B Inhibitors;  ^e^, Mann-Whitney-U test;  ^f^, positive percent values represent an increase in score or dosage from baseline; | | | | | | | | |
