# Supplementary material for: Magnetic Resonance–Guided Focused Ultrasound Thalamotomy May Spare Dopaminergic Therapy in Early‐Stage Tremor‐Dominant Parkinson's Disease: A Pilot Study
Source: Mov Disord. 2022 Aug 29;37(11):2289–95. doi: 10.1002/mds.29200 (PMC9804690; doi:10.1002/mds.29200)
Supplement: Supplementary file 5 — Table S2. characterization of incident Adverse Events observed during the 6‐months follow‐up. [file MDS-37-2289-s004.docx]

| **Supplementary Table 2:** characterization of incident Adverse Events observed during the 6-months follow-up. | | | |
| --- | --- | --- | --- |
|  | **PD-FUS**  **(n=10)** | **PD-ODT**  **(n=20)** | ***p value*** ^e^ |
| **Number of patients with at least one treatment-related AE**^a^ | | | |
| Patients with 1 or more AE | 5 (50%) | 12 (60%) | .706 |
| Patients with 1 or more dopaminergic therapy-related AE | 3 (30%) | 12 (60%) | .245 |
| Patients with 2 or more dopaminergic therapy-related AE | 0 | 3 (15%) | .532 |
| **Type and number of single AEs** | | | |
| **Dopaminergic therapy-related** | | | |
| Motor Fluctuations | 1 (10%) | 3 (15%) | .999 |
| Dyskinesias | 0 | 1 (5%) | .999 |
| Impulse control disorder | 1 (10%) | 2 (10%) | .999 |
| Nausea | 0 | 2 (10%) | .540 |
| Leg edema | 1 (10%) | 1 (5%) | .999 |
| Hypotension | 0 | 3 (15%) | .532 |
| Daytime sleepiness | 0 | 4 (20%) | .272 |
| Anxiety/agitation | 0 | 2 (10%) | .540 |
| Skin reaction to rotigotine | 0 | 2 (10%) | .540 |
| **Thalamotomy Related ^b^** | | | |
| Orofacial paresthesias |  |  |  |
| Transient ^c^ | 1 (10%) | NA | NA |
| Persistent ^d^ | 2 (20%) | NA | NA |
| Ataxia |  |  |  |
| Transient | 4 (40%) | NA | NA |
| Persistent | 0 | NA | NA |
| Hemiparesis |  |  |  |
| Transient | 2 (20%) | NA | NA |
| Persistent | 0 | NA | NA |
| Dysarthria |  |  |  |
| Transient | 1 (10%) | NA | NA |
| Persistent | 0 | NA | NA |
| **MRI or Ultrasonography Related (present only during the MRgFUS procedure)** | | |  |
| Nausea/vomit | 6 (60%) | NA | NA |
| Scalp Numbness | 1 (10%) | NA | NA |
| Dizziness | 6 (60%) | NA | NA |
| Headache | 2 (20%) | NA | NA |
| Uncomfortable heat sensation | 3(30%) | NA | NA |
| Anxiety | 1 (10%) | NA | NA |
| Neck pain | 1 (10%) | NA | NA |
| **Unrelated** | | |  |
| Low back pain | 1 (10%) | 3 (15%) | .999 |
| Prostatic neoplasm | 0 | 1 (5%) | .999 |
| Sleep apnea disorder | 1 (10%) | 0 | .333 |
| Subjective memory loss | 0 | 1 (5%) | .999 |
| Abbreviations: AE, Adverse Event; MRI, Magnetic Resonance Imaging; PD-FUS, Parkinson’s Disease-Focused Ultrasound group; PD-ODT, Parkinson’s Disease-Oral Drug Therapy group; NA, Not Applicable.  ^a^, associated with dopaminergic therapy or thalamotomy;  ^b^, associated with the thalamic lesion;  ^c^, these adverse events were resolved between one to three months after thalamotomy;  ^d^, adverse events which was persistent six months after thalamotomy;  ^e^, Fisher’s exact test. | | | |
